# A WRKY Transcription Factor, *ZmWRKY82*, Conferred Enhanced Drought Stress Tolerance in Maize

**DOI:** 10.3390/plants14192943

**Published:** 2025-09-23

**Authors:** Zhiqiang Wu, Meiyi Liu, Xiangyu Xing, Hanqiao Wang, Dan Li, Xu Fei, Dayong Yang, Peiru Zeng, Wei Yang, Jiabin Ci, Xuejiao Ren, Heng Pan, Liangyu Jiang, Zhenyuan Zang

**Affiliations:** College of Agriculture, Jilin Agricultural University, Changchun 130118, China; 17396774079@163.com (Z.W.); l2932144311@163.com (M.L.); xxy17390949953@126.com (X.X.); wanghanqiao02@163.com (H.W.); 15248522089@163.com (D.L.); 15204380159@163.com (X.F.); 17873869930@163.com (D.Y.); 17785929556@163.com (P.Z.); davidyoung588@126.com (W.Y.); cjb6666@163.com (J.C.); rxj0342@163.com (X.R.); 19187964587@163.com (H.P.)

**Keywords:** maize, drought resilience, *ZmWRKY82*, ROS, ABA

## Abstract

Members of the WRKY transcription factors (TFs) family play crucial roles in biotic and abiotic stress responses in plants, but their roles in response to drought stress in maize (*Zea mays* L.) have not been fully elucidated. Maize *ZmWRKY82*, a group IIc WRKY gene, was isolated from maize using reverse transcription polymerase chain reaction (RT-PCR). Using the UniProt online database, we found that ZmWRKY82 encodes a 222-amino protein with conserved WRKYGKK and C-X_4_-C-X_23_-H-X_1_-H motifs. *ZmWRKY82* is strongly induced by polyethylene glycol (PEG), abscisic acid (ABA), methyl jasmonate (MeJA), salicylic acid (SA), and ethephon (ETH) treatments. The ZmWRKY82 protein was located in the cell nucleus. *ZmWRKY82* had transcriptional activation capability and was able to bind to the W-box element. *ZmWRKY82*-overexpressing *Arabidopsis* and maize exhibited stronger drought resilience, which was associated with enhanced antioxidant enzyme activity and altered transcription level of drought-related genes. These findings suggest that *ZmWRKY82* plays a central role in conferring drought tolerance in maize and may contribute to crop improvement and sustainable agricultural practices.

## 1. Introduction

Drought stress has become more frequent due to global warming and altered rainfall patterns, which severely impair reproductive development, vegetative growth, and yield of maize [1,2]. Drought can reduce corn yields by 39.3%, and severe drought can even result in total crop failure [3]. To mitigate its adverse effects, drought resilience in plants is enhanced by modulating physiological, biochemical, and molecular mechanisms. Such mechanisms include stomatal closure, reduced transpiration rate, osmotic adjustment, increased antioxidant enzyme activity, and regulation of drought-responsive gene expression and transcription factor activities [4,5,6,7]. Investigating drought-resistant genes and breeding resilient cultivars are critical for enhancing crop drought tolerance and productivity.

WRKY transcription factors (TFs) are crucial for mediating plant adaptation to environmental stresses through perceiving external signals and regulating the expression of genes encoding mitigating factors [8,9]. Defined by a highly conserved WRKY domain, the WRKY TF family is divided into three subgroups: I, II, and III. Subgroup I possesses dual WRKY domains, whereas subgroups II and III each feature a single WRKY domain [10]. Structurally, WRKY TFs possess dual conserved structural motifs: WRKYGQK/GKK sequence and zinc finger domain (C-X_4–5_-C-X_22–23_-H-X_1_-H or C-X_7_-C-X_23_-H-X_1_-C). The WRKY TF mediates plant stress adaptation and hormonal signaling via cooperating with the W-box sequence of specific target genes [10,11]. For example, *Paeonia ostii PoWRKY69* is able to increase plant drought resistance in conjunction with the W-box motif of the *PoFBA5* promoter sequence [12]. *AtWRKY63/ABO3* regulates drought resilience in *Arabidopsis* by specifically binding to the W-box cis-regulatory motif of *ABF2* [13]. These findings further demonstrate that WRKY TFs play a critical role in plant responses to various stresses by directly modulating the downstream target gene.

*WRKY82* is an important WRKY TF that participates in diverse stress responses and in the regulation of plant growth and development. The *WRKY82* gene has been identified in multiple plant species, including maize, rice (*Oryza sativa*), mango (*Mangifera indica* L.), and *Gossypium raimondii* [14,15,16,17]. The expression of rice *OsWRKY82* is strongly induced by multiple hormones and stresses, including methyl jasmonate (MeJA), ethephon (ETH), wounding, and high-temperature stress [18]. An elevated *OsWRKY82* transcription level improves rice resistance to bacterial leaf blight by regulating the transcriptional level of defense marker genes [19]. *HbWRKY82*-overexpressing *Hevea brasiliensis* exhibits enhanced drought and salt resilience through coordinately regulating the transcription of stress-responsive genes [20]. These findings suggest that *WRKY82* plays a key role in regulating plant growth and development and in responding to adverse environmental stresses. However, the function of *WRKY82* in maize drought resilience remains unclear.

Through long-term evolution, plants have established inherent defense system to enhance their adaptability to drought stress [1]. These mechanisms include maintaining reactive oxygen species (ROS) homeostasis, increasing abscisic acid (ABA) levels, increasing calcium ion (Ca^2+^) concentration and activating relevant transcription factors [21,22,23]. Among these, ROS are key signaling molecules in plant stress responses to external stress [24]. An increasing number of studies suggest that WRKY TFs play a role in regulating ROS homeostasis in plants. For example, overexpression of cotton *GhWRKY68* diminishes tobacco’s drought and salt resilience by promoting the ROS accumulation [25]. Cotton *GhWRKY17* negatively regulates the drought and salt resilience of cotton by increasing ROS accumulation and diminishing the transcription level of ROS scavenger genes [26]. Sweet potato *IbWRKY2*-overexpressing *Arabidopsis* exhibits greater drought and salt resilience, as well as a lower ROS content [27]. *Brachypodium distachyon BdWRKY36*-overexpressing tobacco exhibits stronger resilience to drought and lower ROS contents [28]. These findings suggest that WRKY TFs can increase plant drought resilience through regulating ROS accumulation.

ABA, a key signaling molecule, plays central roles in mediating plant responses to drought stresses [29]. Many studies show that WRKY TFs mediate plant drought resilience by regulating the ABA signaling pathway [30]. *WRKY57*-overexpressing *Arabidopsis* exhibits stronger drought resilience and elevated endogenous ABA level [31]. Loss of function of *GhWRKY21* improves cotton drought resilience, mainly by regulating the ABA signaling pathway [32]. Elevated expression of *Myrothamnus flabellifolia MfWRKY17* markedly improves *Arabidopsis* resilience to drought stresses via regulating ABA biosynthesis and the transcriptional level of stress-responsive genes [33]. The *GsWRKY20* gene from *Glycine soja* increases *Arabidopsis* drought resilience through modulating the closure of stomata and engaging the ABA-dependent signaling pathway [34]. Therefore, WRKY TFs can regulate ABA biosynthesis and signal transduction genes to mediate plant drought resistance.

Although more than 100 unique WRKY TFs have been identified and characterized in maize [35], their drought resilience function has not been fully elucidated. In this study, we identified a WRKY gene that was significantly induced in maize under drought stress based on transcriptome data, designated as *ZmWRKY82*. To investigate its function in the drought stress response, we analyzed its expression pattern and subcellular localization, along with the physiological indicators and phenotypes of *ZmWRKY82*-overexpressing plants, after drought stress treatment. This study offers a foundation for advancing analysis of WRKY TFs in maize drought response and is critical for the development of novel drought-tolerant cultivars, ensuring food security and sustainable agriculture.

## 2. Results

### 2.1. Cloning and Characterization of ZmWRKY82

We cloned the *ZmWRKY82* gene from maize B73 (a maize inbred line). The coding sequence (CDS) of *ZmWRKY82* was 666 base pairs long, encoding a protein of 222 amino acids, possessing a predicted molecular mass of 22.96 kDa and a theoretical isoelectric point (pI) of 6.65. Homologous amino acid sequences in *Oryza sativa* L., *Arabidopsis thaliana*, and *Sorghum bicolor* were searched using the phytozome BLAST online tool (https://phytozome-next.jgi.doe.gov/blast-search, accessed on 21 September 2025). Phylogenetic analysis reveals that ZmWRKY82 is closely related to SbWRKY83 (a group IIc WRKY gene) (Figure S1a) [36]. Therefore, ZmWRKY82 is a member of WRKY TF group IIc. Multiple sequence alignment of amino acid homologs demonstrated that ZmWRKY82 contains a (C-X_4_-C-_X23_-H-X_1_-H) zinc finger sequence and a conserved WRKY (WRKYGKK) domain (Figure S1b). These results suggest that *ZmWRKY82* is a member of the WRKY TF family and may have the ability to bind the W-box sequence.

### 2.2. Expression Pattern of ZmWRKY82 in Maize Under Different Stresses

**Figure 1:**
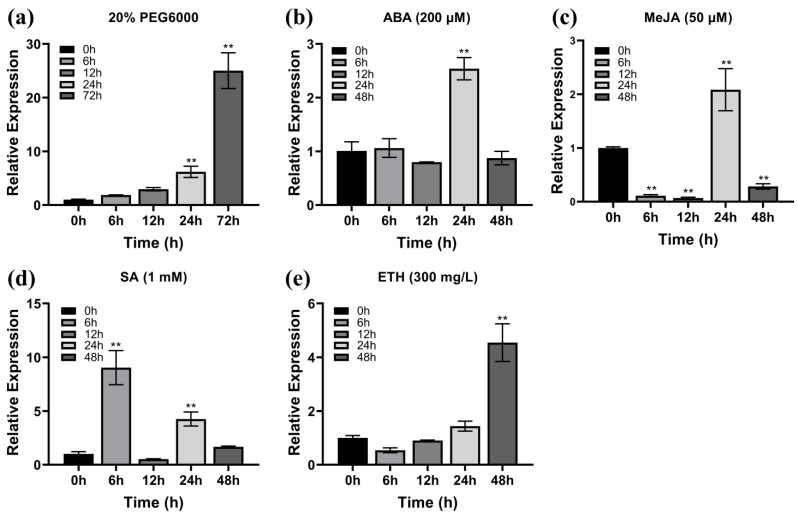
The relative expression level of *ZmWRKY82* under various treatments: (**a**) 20% PEG6000 treatment, (**b**) 200 μM ABA treatment, (**c**) 50 μM MeJA treatment, (**d**) 1 mM SA treatment and (**e**) 300 mg/L ETH treatment. *ZmTub* (GRMZM2G066191) was used as an endogenous reference for data standardization. The relative expression levels were analyzed using the 2^−ΔΔCT^ method. 0 h is set as the control. The significance analysis compared with 0 h was performed using one-way ANOVA (** *p* < 0.01). Bars indicate standard error of the mean. The experiment was performed using three biological replicates from three independent plants.

Previous studies have shown that the transcription levels of WRKY TFs are increased by stress treatments with polyethylene glycol (PEG), ABA, MeJA, salicylic acid (SA), and ETH [37,38,39]. To explore the signaling pathways in which *ZmWRKY82* may be involved, maize seedlings at the third-leaf stage (V3) were subjected to various hormone and stress treatments, including irrigation with PEG solution or spraying with ABA, MeJA, SA, and ETH solution. The treated seedlings were cultured in an incubator at 28 °C (16 h light/8 h dark cycle). The transcription level of *ZmWRKY82* in the V3- stage maize seedling leaves was analyzed with quantitative real-time polymerase chain reaction (qRT-PCR). The transcription level of *ZmWRKY82* progressively increased following treatment with 20% PEG6000, reaching its maximum at 72 h (25-fold increase) (Figure 1a). After 200 μM ABA treatment, the transcription level of *ZmWRKY82* was notably increased and reached a maximum at 24 h (2.5-fold increase) (Figure 1b). After treatment with 50 μM MeJA, the transcription level of *ZmWRKY82* decreased significantly between 6 h and 12 h, followed by a sharp increase, peaking at 24 h (Figure 1c). The transcription level of *ZmWRKY82* was significantly upregulated at 6 h after 1 mM SA treatment, reaching a value 9 times higher than that at 0 h (Figure 1d). After 300 mg/L ETH treatment, the transcription level of *ZmWRKY82* was down regulated at 6 h and 12 h, followed by an upward trend, and reached its peak at 48 h (Figure 1e). The results show that the *ZmWRKY82* gene is induced by PEG, ABA, MeJA, SA, and ETH, with the highest transcription level observed after treatment with 20% PEG6000. Thus, we hypothesize that *ZmWRKY82* participates in multiple signaling pathways and contributes to the plant’s response to drought stress.

### 2.3. Subcellular Localization of ZmWRKY82

**Figure 2:**
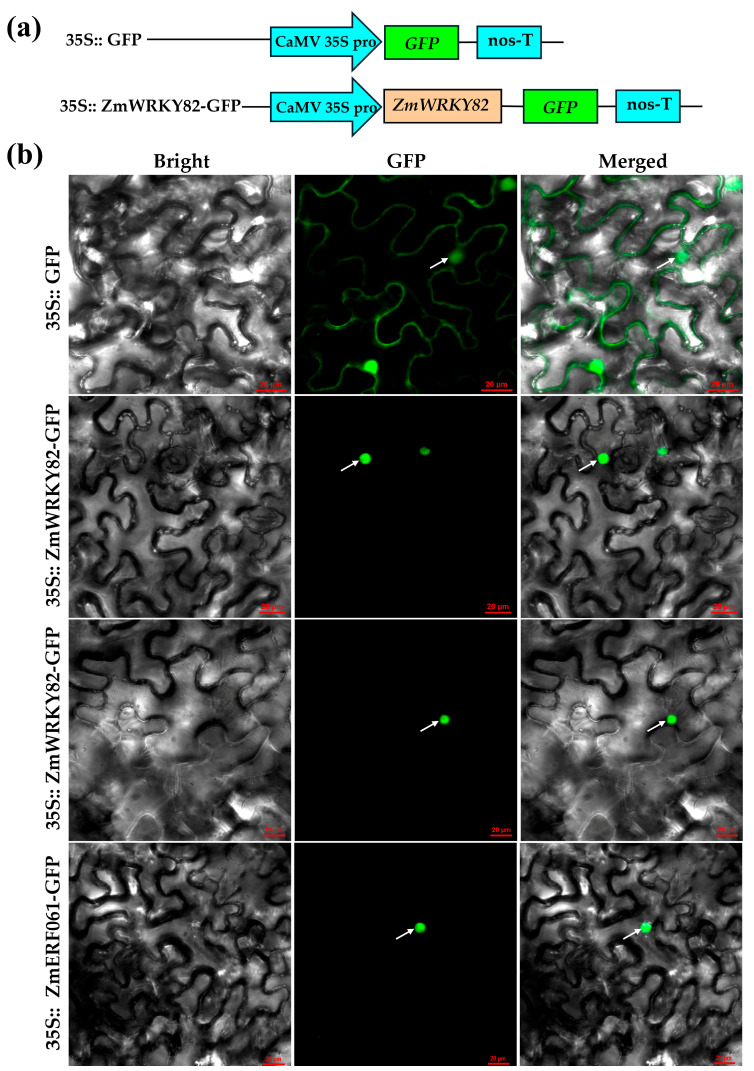
Subcellular location of ZmWRKY82-GFP in the epidermal cells of *Nicotiana benthamiana* leaves. (**a**) Schematic diagram of pCAMBIA1302-GFP (35S:: GFP) and the pCAMBIA1302-ZmWRKY82-GFP (35S:: ZmWRKY82-GFP). (**b**) ZmWRKY82-GFP, pCAMBIA1302-GFP (35S:: GFP) and ZmERF061-GFP were transiently expressed in the epidermal cells of tobacco leaves, respectively. The white arrow indicated the nucleus of tobacco epidermal cells. Scale bars = 20 μm.

WRKY TF, which acts as a transcriptional activation factor, is commonly localized to the nucleus [40]. Following the method of Zang et al. (2021) [41], we removed the stop codon of ZmWRKY82 CDS and ligated ZmWRKY82 CDS into pCAMBIA1302 to construct the pCAMBIA1302-ZmWRKY82-GFP (35S:: ZmWRKY82-GFP) recombinant vector (Figure 2a). The 35S:: ZmWRKY82-GFP recombinant vector was introduced into tobacco leaves via *Agrobacterium*-mediated transformation. As shown in Figure 2b, the fluorescence signal of pCAMBIA1302-GFP (35S:: GFP) is detected in both the nucleus and cell membrane. However, 35S:: ZmWRKY82-GFP was detected exclusively in the nucleus, which is consistent with nuclear-localized protein ZmERF061 [41]. This result indicates that ZmWRKY82 may exert transcriptional activation function in the nucleus.

### 2.4. ZmWRKY82 Has Transcriptional Activation Potential and Specifically Binds to W-Box Elements

**Figure 3:**
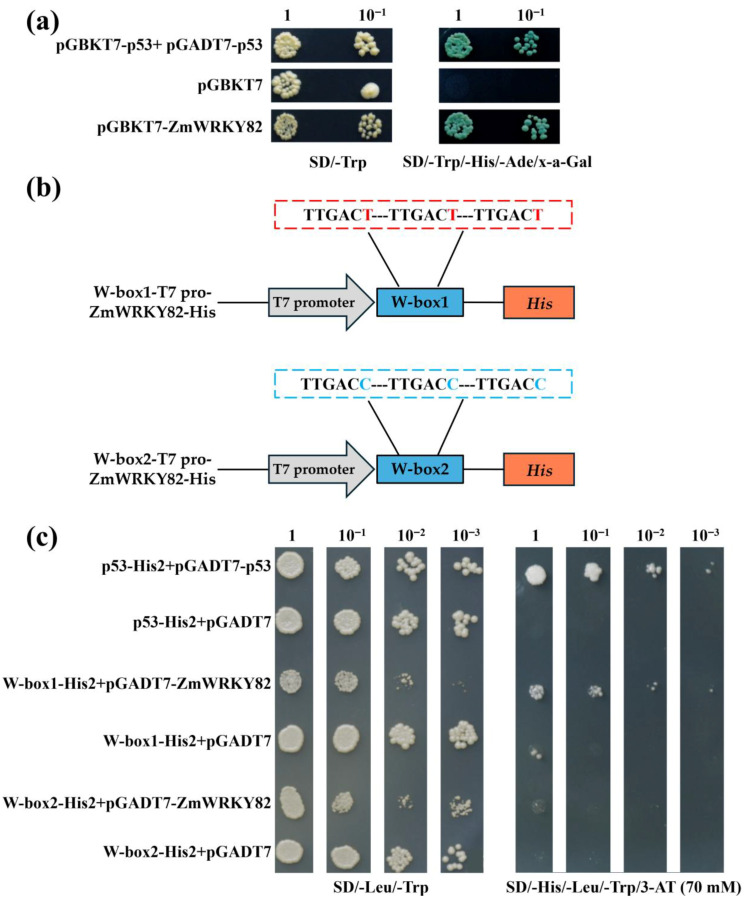
Transcription activity and DNA-binding activity analysis of ZmWRKY82. (**a**) Transcriptional activation analysis of ZmWRKY82. (**b**) Sequence of the triple tandem repeats of the W-box1 and W-box2. Schematic diagram of W-box1 (TTGACT)-T7 pro-ZmWRKY82-His and W-box2 (TTGACC)-T7 pro-ZmWRKY82-His constructs. (**c**) The DNA-binding activity of ZmWRKY82 was assessed via yeast one-hybrid assay using 3× W-box1 or 3× W-box2 as bait. Positive control p53-His2+pGADT7-p53, negative control p53-His2+pGADT7, W-box1-His2+pGADT7-ZmWRKY82, W-box1-His2+pGADT7, W-box2-His2-+pGADT7-ZmWRKY82, W-box2-His2+pGADT7 were grown on SD/-Leu/-Trp medium or SD/-His/-Leu/-Trp with 3-AT (70 mM) medium.

To further study the transcriptional activation potential of *ZmWRKY82*, the recombinant construct of the pGBKT7-ZmWRKY82 was created. pGBKT7-ZmWRKY82 recombinant construct plasmid was transformed into Y2HGold yeast cells. After 3 days (d) of cultivation, all the yeast cells grew well on SD/-Trp medium (synthetic dextrose minimal medium lacking tryptophan) (Figure 3a). In contrast, robust growth and blue coloration on SD/-Trp/-His/-Ade/X-α-Gal medium (synthetic dextrose minimal medium lacking tryptophan, histidine, and adenine, supplemented with α-galactosidase) were exclusively observed in yeast cells carrying pGBKT7-ZmWRKY82 or the pGBKT7-53+pGADT7-T (positive control) plasmids (Figure 3a). These findings suggest that *ZmWRKY82* possesses transcriptional activation activity in the yeast expression system.

Subsequently, we created the pGADT7-ZmWRKY82 recombinant construct to verify whether *ZmWRKY82* could bind to the W-box sequence. The recombinant reporter plasmid pHis2 was constructed to contain three tandem repeats of the W-box1 (TTGACT) and W-box2 (TTGACC) sequences (Figure 3b). The recombinant reporter plasmids, along with either the pGADT7-ZmWRKY82 or pGADT7, were introduced into the Y187 yeast cells. Following a 3 d incubation, all yeast transformants exhibited normal growth on SD/-Leu/-Trp medium (synthetic dextrose minimal medium lacking leucine and tryptophan) (Figure 3c). Y187 [p53-His2+pGADT7-p53] (positive control), Y187 [W-box1-His2+pGADT7-ZmWRKY82], and Y187 [W-box2-His2+pGADT7-ZmWRKY82] yeast cells were able to grow on SD/-His/-Leu/-Trp/3-AT (70 mM) medium (a synthetic dextrose minimal medium lacking histidine, leucine, and tryptophan, supplemented with 70 mM 3-AT) (Figure 3c). However, the yeast cells of Y187 [p53-His2+pGADT7] (negative control), Y187 [W-box1-His2+pGADT7], and Y187 [W-box2-His2+pGADT7] did not grow or grew poorly on SD/-His/-Leu/-Trp/3-AT (70 mM) medium (Figure 3c). These findings suggest that *ZmWRKY82* specifically interacts with the W-box (TTGACT/C) motif.

### 2.5. Overexpression of ZmWRKY82 Increases Arabidopsis Resilience to Drought Stress

**Figure 4:**
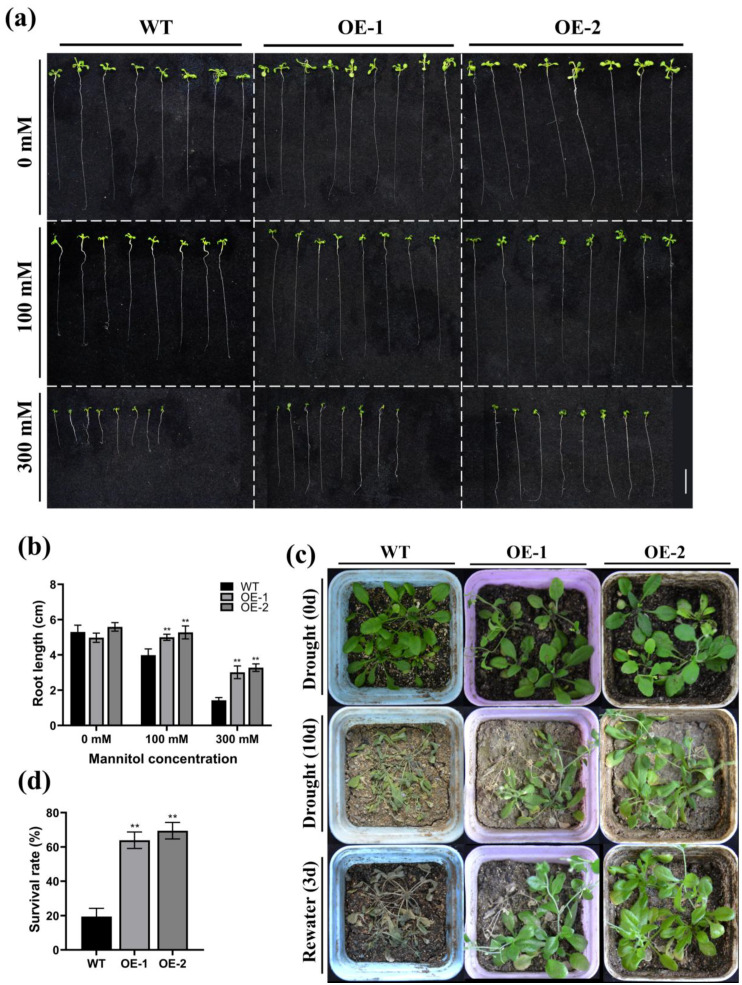
Overexpression of *ZmWRKY82* enhances the drought tolerance of *Arabidopsis*. (**a**) Root lengths of *ZmWRKY82*-overexpressing lines (OE-1 and OE-2) and wild-type (WT) *Arabidopsis* (Columbia) seedlings were measured on control conditions (1/2 MS medium) and on 1/2 MS medium supplemented with 100 mM or 300 mM mannitol. Scale bars = 1 cm. (**b**) Root lengths of *ZmWRKY82*-overexpressing lines and WT were analyzed under 1/2 MS medium, 100 mM or 300 mM mannitol treatment. (**c**) The 21-day-old plants were subjected to water withholding treatment for 10 d, and then re-watering for 3 d. (**d**) The survival rate was analyzed after re-watering for 3 d. Each biological replicate in the drought stress experiment comprised 12 plants. The survival rate for each biological replicate was calculated as the mean value of 12 plants. The graphed data were the average (±SD) of three independent biological replicates. The significance analysis compared with WT was performed using one- and two-way ANOVAs (** *p* < 0.01). The photos show representative plants.

Mannitol-induced osmotic stress is commonly used to assess *Arabidopsis thaliana*’s drought tolerance [42]. To investigate the drought resilience of *ZmWRKY82* in depth, we subjected wild-type (WT) *Arabidopsis* (Columbia) and T3-generation-overexpressing *Arabidopsis* strains (OE-1 and OE-2) to mannitol-induced osmotic stress (Figure S2a,b). No notable variation in root length was observed among the WT, OE-1, and OE-2 strains when grown on medium without mannitol for 14 d (Figure 4a,b). The root lengths of the OE-1 and OE-2 strains were notably higher than in the WT plants when grown on medium containing 100 mM or 300 mM mannitol for 14 d (Figure 4a,b). From these results, we hypothesized that *ZmWRKY82* enhances the resilience of *Arabidopsis* to mannitol-induced osmotic stress.

The 21-day-old WT, OE-1, and OE-2 strains were subjected to 10 d of water withholding treatment. After this treatment and 3 d of re-watering, WT plants exhibited more severe wilting compared with the OE-1 and OE-2 plants (Figure 4c). Analysis showed that 63.89% of the OE-1 strain, 69.44% of the OE-2 strain, and 19.44% of the WT strain survived (Figure 4d). The results demonstrate that *ZmWRKY82* can increase *Arabidopsis* drought resilience.

### 2.6. Overexpression of ZmWRKY82 Increases Arabidopsis Drought Resilience by Affecting Antioxidant Enzyme Activity

**Figure 5:**
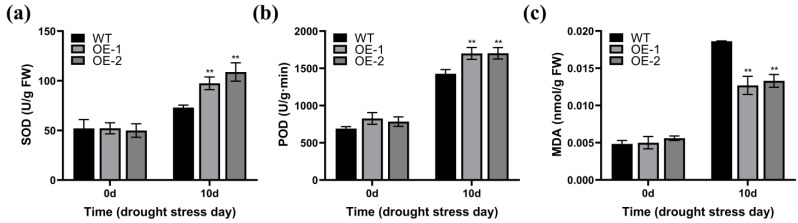
Determination of physiological indices in the leaf tissue of wild-type (WT) *Arabidopsis* (Columbia) and *ZmWRKY82*-overexpressing *Arabidopsis* at 0 d and after 10 d of water withholding treatment. (**a**) SOD activity, (**b**) POD activity and (**c**) MDA content in *ZmWRKY82*-overexpressing *Arabidopsis* and WT plants before and after 10 d of water withholding treatment. Enzyme activities were expressed as enzyme units per gram fresh weight (U/g FW). The experiments were performed with three biological replicates. The data were the average (±SD) of three independent experiments. The significance analysis compared with WT was performed using two-way ANOVA (** *p* < 0.01).

We also assessed the relevant physiological parameters in the leaf tissue of the WT, OE-1, and OE-2 *Arabidopsis* strains at 0 d and after 10 d of water withholding treatment. No notable differences were observed in superoxide dismutase (SOD) and peroxidase (POD) activity, and malondialdehyde (MDA) content among the WT, OE-1, and OE-2 plants before water withholding treatment (Figure 5a–c). After 10 d of this treatment, the OE-1 and OE-2 transgenic strains showed increased SOD and POD activity and reduced MDA level compared to the WT strains (Figure 5a–c). These findings indicate that *ZmWRKY82* may increase *Arabidopsis* drought resilience by increasing SOD and POD activity and decreasing MDA.

### 2.7. Overexpression of ZmWRKY82 Increases Arabidopsis Drought Resilience by Affecting Defense Gene Expression

**Figure 6:**
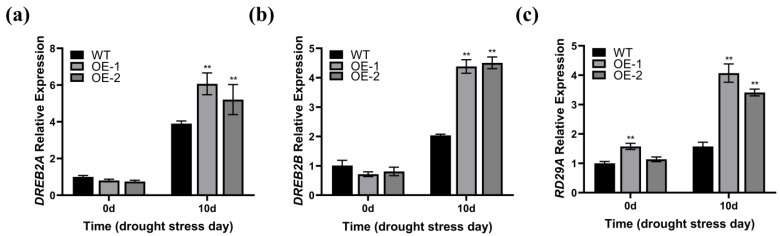
Determination of transcription level of drought-stress responsive genes in the leaf tissue of wild-type (WT) *Arabidopsis* (Columbia) and *ZmWRKY82*-overexpressing *Arabidopsis* at 0 d and after 10 d of water withholding treatment. Expression analysis of (**a**) *DREB2A* (AT5G05410), (**b**) *DREB2B* (AT3G11020) and (**c**) *RD29A* (AT5G52310) drought-stress responsive genes in *ZmWRKY82*-overexpressing *Arabidopsis* and WT lines at 0 d and after 10 d of water withholding treatment. *ACTIN2* (At3g18780) was used as an endogenous reference for data standardization. The experiments were performed with three biological replicates. The data were the average (±SD) of three independent experiments. The significance analysis compared with WT was performed using two-way ANOVA (** *p* < 0.01).

We further measured the transcription level of the drought-induced genes *DREB2A*, *DREB2B*, and *RD29A* [43,44] in the leaf tissue of the WT and *ZmWRKY82*-overexpressing *Arabidopsis* strains at 0 d and after 10 d of water withholding treatment. Prior to water withholding treatment, no notable discrepancies were observed in the transcription levels of *DREB2A* and *DREB2B* among the OE-1, OE-2, and WT strains (Figure 6a,b). The transcription level of *RD29A* was notably up-regulated in the OE-1 and OE-2 strains before treatment (Figure 6c). The transcription level of the drought-induced genes was notably elevated in the OE-1 and OE-2 strains compared to the WT after 10 d of water withholding treatment (Figure 6a–c). These results suggest that *ZmWRKY82* may modulate the transcription level of these genes, thereby contributing to the drought stress response in *Arabidopsis*.

### 2.8. Overexpression of ZmWRKY82 Enhances Drought Resilience in Maize

**Figure 7:**
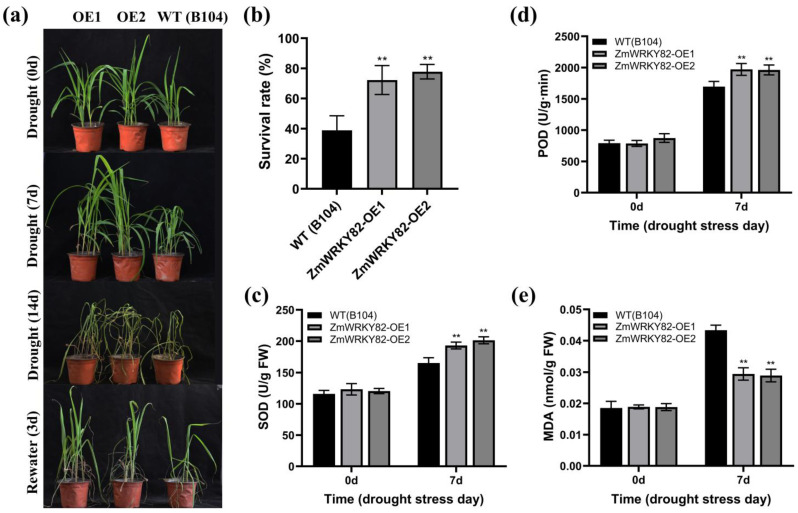
Overexpression of *ZmWRKY82* enhanced the drought tolerance of maize. (**a**) The phenotype in *ZmWRKY82*-overexpressing maize lines and WT (B104) lines at 0 d, after 14 d of water withholding treatment, and after 3 d of re-watering. (**b**) The survival rate was analyzed by re-watering for 3 d. (**c**) SOD activity, (**d**) POD activity and (**e**) MDA content in the leaf tissue of *ZmWRKY82*-overexpressing maize lines and WT (B104) lines were assessed at 0 d and after 7 d of water withholding treatment. Enzyme activities were expressed as enzyme units per gram fresh weight (U/g FW). The experiments were performed with three biological replicates. The data were the average (±SD) of three independent experiments. The significance analysis compared with was performed using one- and two-way ANOVAs (** *p* < 0.01). Each biological replicate in the drought stress experiment comprised 12 plants.

To further research the drought resilience performance of *ZmWRKY82* in maize, we subjected the maize inbred line B104 and *ZmWRKY82*-overexpressing maize strains (*ZmWRKY82*-OE1 and *ZmWRKY82*-OE2) to drought stress treatment. All maize strains exhibited similar growth before water withholding treatment (Figure 7a). After 7 d and after 14 d of water withholding treatment, WT (B104) exhibited more severe wilting compared with the *ZmWRKY82*-overexpressing maize strains. After 3 d of re-watering, the *ZmWRKY82*-overexpressing maize strains exhibited better growth compared with the WT (B104). The survival rates of *ZmWRKY82*-OE1 and *ZmWRKY82*-OE2 were 72.22% and 77.78%, respectively, whereas WT (B104) showed a survival rate of only 38.89% (Figure 7b). These findings indicate that *ZmWRKY82* enhances drought resilience in maize plants.

We assessed the physiological parameters in the leaf tissue of the WT (B104) and *ZmWRKY82*-overexpressing maize strains at 0 d and after 7 d of water withholding treatment. No notable discrepancies were observed in SOD and POD activity and MDA content among all maize strains before water withholding treatment (Figure 7c–e). The SOD and POD activity of the *ZmWRKY82*-OE1 and *ZmWRKY82*-OE2 strains was notably higher compared to the WT (B104) strains after 7 d of water withholding treatment (Figure 7c,d). The MDA levels of the *ZmWRKY82*-OE1 and *ZmWRKY82*-OE2 strains were notably lower than those of the WT (B104) strains after 7 d of treatment (Figure 7e). These findings demonstrate that *ZmWRKY82* positively regulates drought resilience in maize by increasing SOD and POD activity and decreasing the MDA level.

### 2.9. ZmWRKY82 Affects the Transcriptional Level of Many Drought-Related Genes

**Figure 8:**
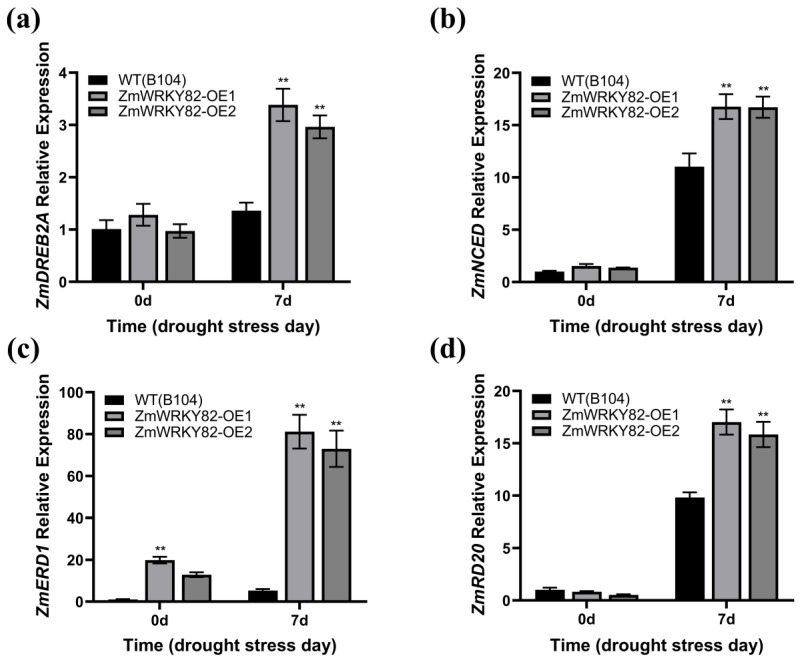
The relative expression level of the drought-stress-responsive genes were analyzed in the leaf tissue of WT (B104) lines and *ZmWRKY82*-overexpressing maize lines at 0 d and after 7 d of water withholding treatment, including: (**a**) *ZmDREB2A* (GRMZM2G006745). (**b**) *ZmNCED* (GRMZM5G838285). (**c**) *ZmERD1* (GRMZM2G172230). (**d**) *ZmRD20* (GRMZM2G342685). *ZmTub* (GRMZM2G066191) was used as an endogenous reference for data standardization. The experiments were performed with three biological replicates. The data were the average (±SD) of three independent experiments. The significance analysis compared with WT was performed using two-way ANOVA (** *p* < 0.01).

The transcription level of drought-related genes *ZmDREB2A*, *ZmNCED*, *ZmERD1*, and *ZmRD20* [45] was assessed in the leaf tissue of WT (B104) and *ZmWRKY82*-overexpressing maize strains at 0 d and after 7 d of water withholding treatment. No notable differences were observed in the transcription levels of *ZmDREB2A*, *ZmNCED*, and *ZmRD20* among the *ZmWRKY82*-OE1, *ZmWRKY82*-OE2, and WT (B104) strains before water withholding treatment (Figure 8a,b,d). The transcription level of *ZmERD1* was notably higher in *ZmWRKY82*-overexpressing maize strains compared to WT (B104) at 0 d of water withholding treatment (Figure 8c). The transcription level of *ZmDREB2A*, *ZmNCED*, *ZmERD1*, and *ZmRD20* in the *ZmWRKY82*-overexpressing maize strains is substantially higher than in the WT (B104) strains after 7 d of water withholding treatment (Figure 8a–d). These findings suggest that *ZmWRKY82* improves drought resilience in maize via regulating the transcription level of drought response genes.

### 2.10. Overexpression of ZmWRKY82 Increases Sensitivity to ABA in Maize

**Figure 9:**
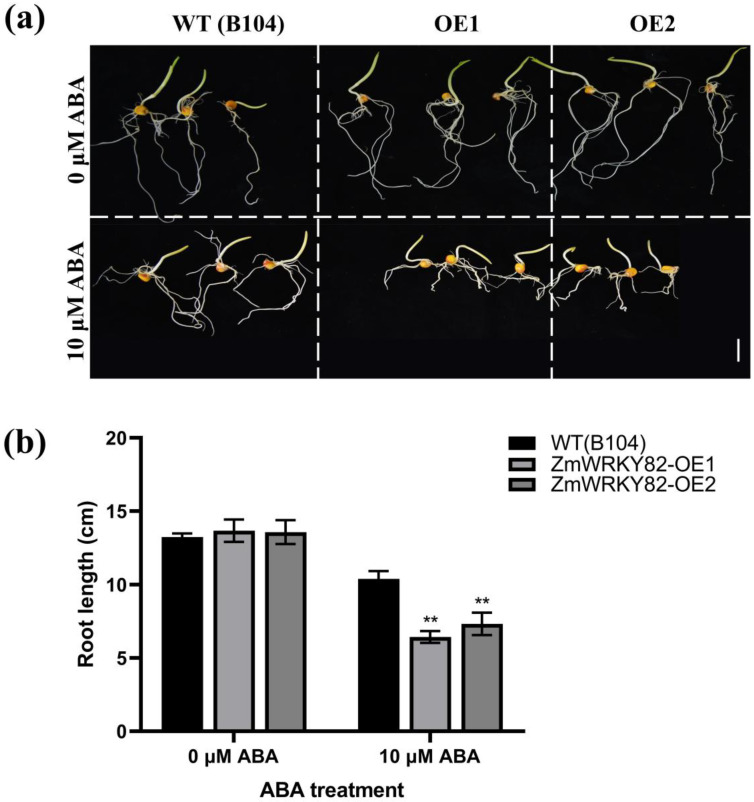
Root lengths of *ZmWRKY82*-overexpressing maize lines were significantly longer under 10 μM ABA treatment with WT (B104). (**a**) Phenotype of root length in *ZmWRKY82*-overexpressing maize lines (*ZmWRKY82*-OE1 and *ZmWRKY82*-OE2) and WT (B104) under 0 μM or 10 μM ABA treatments. Scale bars = 2.5 cm. (**b**) Root lengths of *ZmWRKY82*-overexpressing maize lines under 0 μM or 10 μM ABA treatment. The experiments were performed with three biological replicates. The data were the average (±SD) of three independent experiments. The significance analysis compared with WT was performed using two-way ANOVA (** *p* < 0.01).

To further confirm the response of *ZmWRKY82* to ABA treatment, WT (B104) and *ZmWRKY82*-overexpressing maize seeds were treated with exogenous ABA. Root length was inhibited to varying degrees in all seeds after 4 d of treatment, but the *ZmWRKY82*-overexpressing maize seeds exhibited notably shorter roots compared to the WT (B104) (Figure 9a,b). These findings indicate that the overexpression of *ZmWRKY82* increases maize sensitivity to ABA.

## 3. Discussion

Drought is a notable environmental stressor for plants, and severe drought stress causes irreversible damage to plants [46,47]. The literature suggests that WRKY TFs are crucial in regulating plant responses to drought stress [48], but the function in maize is not yet fully understood. In this study, we found that the TF *ZmWRKY82* plays a valuable role in enhancing drought resilience in maize and *Arabidopsis*.

Much research has shown that WRKY TFs play a key role in mediating plant responses to drought stress [49]. In this study, the transcription level of *ZmWRKY82* was significantly increased in response to 20% PEG 6000 treatment (Figure 1a), and overexpression of *ZmWRKY82* positively regulated drought resilience of *Arabidopsis* and maize plants (Figure 4 and Figure 7). These findings demonstrate that *ZmWRKY82* increases drought resilience of *Arabidopsis* and maize. Most WRKY TF members possess transcriptional activation potential and bind to the W-box sequence [49]. In this study, we found that *ZmWRKY82* exhibits transcriptional activation activity (Figure 3a) and can bind the W-box (TTGACT/C) sequence (Figure 3c). Therefore, we hypothesize that *ZmWRKY82* may enhance plant drought resistance by binding to W-box elements in the promoters of drought-responsive genes and activating their expression.

ROS are important messengers in the plant defense system, and numerous studies have demonstrated that WRKY TFs are closely associated with ROS accumulation in response to drought stress in plants [50]. Overexpression of yellowhorn *XsWRKY20* positively regulated drought resilience of tobacco by maintaining ROS homeostasis [51]. *ZmWRKY79* improved drought resilience in *Arabidopsis* via increasing antioxidant enzyme activity, decreasing MDA content and promoting ROS scavenging [42]. In our study, the SOD and POD activity was notably higher in *ZmWRKY82*-overexpressing *Arabidopsis* and maize strains than in the WT strains following water withholding treatment (Figure 5a,b and Figure 7c,d). The MDA content was notably lower in *ZmWRKY82*-overexpressing *Arabidopsis* and maize strains compared to the WT strains (Figure 5c and Figure 7e). Our results suggest that elevated *ZmWRKY82* expression may enhance the ROS scavenging capacity of plants and mitigate drought-induced cellular damage.

The ABA signaling pathway in plants plays a key regulatory role in defense signaling pathways [52]. Previous studies have reported that *TaWRKY1* boosts drought resilience in *Arabidopsis* through participating in the ABA-dependent pathway [53]. Elevated expression of *TaWRKY31* positively regulates the drought resilience of wheat by adjusting the transcription level of ABA-responsive genes [54]. Overexpression of *OsWRKY45* positively regulates the drought resilience of *Arabidopsis* via modulating the transcription level of ABA response genes [55]. Our findings suggest that ABA treatment notably up-regulated the transcription level of *ZmWRKY82* (Figure 1b), and *ZmWRKY82*-overexpressing maize plants displayed enhanced sensitivity to exogenous ABA (Figure 9). Our results are consistent with other reports, suggesting that *ZmWRKY82* may play a key role in regulating maize drought resilience via the ABA signaling pathway.

## 4. Materials and Methods

### 4.1. Plant Material, Growing Conditions, and Treatments

Both the maize seeds (*Zea mays* inbred lines B73 and B104) and *Arabidopsis* WT seeds were provided by the Maize Breeding Innovation Team at Jilin Agricultural University. The maize seeds were germinated in an incubator at 28 °C. The maize seedlings were then transplanted into a 1:1 (*v*/*v*) mixture of sterile nutrient soil and vermiculite and subsequently cultivated under a 16 h light/8 h dark photoperiod at 28 °C until reaching the V3-stage. Soil moisture was maintained at 80% of field capacity. For PEG treatment, the V3-stage maize plants were placed in containers and irrigated once with 100 mL of a 20% (*w*/*v*) PEG6000 solution until the soil was saturated, with water treatment serving as the control. For phytohormone treatments, water treatment (control), 200 μM ABA, 50 μM MeJA, 1 mM SA, and 300 mg/L ETH were sprayed on the leaves of V3-stage maize plants. Treated leaves (similar growth patterns) from three independent plants were collected at 0 h, 6 h, 12 h, 24 h, 48 h, or 72 h and stored at −80 °C.

### 4.2. RNA Extraction and qRT-PCR Analysis

Total RNA was extracted from maize leaf tissue using the phenol/chloroform-based TRIzol reagent (Tiangen, Beijing, China). Total cDNA was obtained from the RNA using a ReverTra Ace^®^ qPCR RT kit (TOYOBO, Shanghai, China). The maize gene *ZmTub* (GRMZM2G066191) and the *Arabidopsis* gene *ACTIN2* (At3g18780) were used as internal controls. Three independent biological replicates were conducted for the experiment. Relative transcript abundance was determined using the 2^−∆∆CT^ method [56]. The sequences of all primers are provided in Table S1.

### 4.3. Subcellular Localization 

The ZmWRKY82-GFP recombinant construct was generated by inserting the *ZmWRKY82* CDS into the *Nco* I and *Spe* I restriction enzyme sites of pCAMBIA1302 using the Seamless Cloning Kit (In-Fusion cloning) (Beyotime, Shanghai, China). The sequences of (ZmWRKY82-1302) primer are provided in Table S1. *Agrobacterium* competent cells GV3101 (pSoup-p19) (Coolaber, Beijing, China) were transformed with the ZmWRKY82-GFP, pCAMBIA1302, and ZmERF061 plasmids according to the manufacturer’s instructions. *Agrobacterium* carrying ZmWRKY82-GFP, pCAMBIA1302, or ZmERF061 was separately introduced into 28-day-old tobacco leaves via an *Agrobacterium*-mediated transformation approach [57]. Inoculated tobacco leaves were incubated in the dark at 22 °C for 24 h. The green fluorescent signal in tobacco leaves was visualized using confocal laser scanning microscopy (CLSM) (ZEISS, Shanghai, China) at 488 nm.

### 4.4. Transcription Activity Assay of ZmWRKY82

The pGBKT7-ZmWRKY82 recombinant vector was constructed by inserting the full-length *ZmWRKY82* CDS into the *Nco* I and *EcoR* I restriction sites of pGBKT7 using the In-Fusion cloning method. The sequences of (ZmWRKY82-pGBKT7) primer are provided in Table S1. Three transformation mixtures, including pGBKT7-p53+pGADT7-p53, pGBKT7, and pGBKT7-zmWRKY82, were transformed into the Y2HGold yeast strain (Coolaber, Beijing, China). Yeast transformants were cultured on SD/-Trp medium and SD/-Trp/-His/-Ade medium supplemented with X-α-Gal and incubated at 28 °C for 3 d.

### 4.5. Yeast One-Hybrid Assay

To generate the pGADT7-ZmWRKY82 recombinant construct, the full-length *ZmWRKY82* CDS was inserted into the *EcoR* I and *BamH* I restriction sites of pGADT7 vector using the In-Fusion cloning method. The sequences of (ZmWRKY82-pGADT7) primers are provided in Table S1. The sequences of W-box1 (TTG ACT) and W-box2 (TTG ACC) were ligated into pHis2 vectors by repeating the tandem three times. According to the instructions provided with the Y187-pHis2 Yeast One-Hybrid Interaction Verification Kit (Coolaber, Beijing, China), the four plasmid combinations W-box1-His2+pGADT7-ZmWRKY82, W-box1-His2+pGADT7, W-box2-His2+pGADT7-ZmWRKY82, and W-box2-His2+pGADT7 were cotransformed into Y187 competent cells, respectively. The yeast cells were inoculated into SD/-Leu/-Trp yeast medium for 3 d (30 °C). Then, the yeast colonies were subjected to serial dilutions (1:1, 1:10, 1:100, and 1:1000) and inoculated into SD/-Leu/-Trp and SD/-His/-Leu/-Trp/3-AT (70 mM) medium, followed by incubation for 3 d (30 °C).

### 4.6. Creation of Transgenic Arabidopsis and Maize

pCAMBIA3301-35S-ZmWRKY82 expression construct was generated by inserting the full-length *ZmWRKY82* CDS into the *Pml* I and *Nco* I restriction sites of pCAMBIA3301 using the In-Fusion cloning method. The sequences of (ZmWRKY82-35S) primers are provided in Table S1. Positive transgenic plants were generated using flower inflorescence immersion method [58].

The full-length CDS of *ZmWRKY82* was ligated into the pCAMBIA3301-UBI vector using T4 DNA ligase (TaKaRa, China) to generate the pCAMBIA3301-UBI-ZmWRKY82 expression construct. The pCAMBIA3301-UBI-ZmWRKY82 expression vector was transformed into *Agrobacterium tumefaciens* strain EHA105 (Coolaber, Beijing, China). The *Agrobacterium* strain carrying pCAMBIA3301-UBI-ZmWRKY82 was introduced into maize inbred line B104 via the *Agrobacterium*-mediated transformation method [59]. The positive transgenic strains were confirmed using glufosinate screening, Bar diagnostic strips, and qRT-PCR analysis. The sequences of the (ZmWRKY82-UBI and ZmWRKY82-Q) primers are provided in Table S1.

### 4.7. Drought and ABA Tolerance Assay

For the mannitol osmotic stress assay [42], *ZmWRKY82*-overexpressing *Arabidopsis* and WT *Arabidopsis* seeds were germinated on 1/2 MS medium supplemented with mannitol (0 mM, 100 mM, and 300 mM) for 14 d (16 h light/8 h dark cycle, 22 °C), and their root length was observed.

For the drought stress assay [60], WT *Arabidopsis* and T3 generation *ZmWRKY82*-overexpressing *Arabidopsis* strains were cultured on 1/2 MS medium for 7 d. Planter boxes were filled to three-quarters of their capacity with a 1:1 (*v*/*v*) mixture of sterile nutrient soil and vermiculite. Subsequently, the seedlings were transplanted into planter boxes and grown for 14 d (16 h light/8 h dark cycle, 22 °C). The planter boxes were placed in a propagation tray and watered every 3 d to maintain soil moisture at 80% of field capacity. Subsequently, the plants were subjected to water withholding treatment for 10 d until the soil moisture reached 30% of field capacity. The plants were then subjected to a 3 d re-watering treatment, maintaining soil moisture at 80% of field capacity. The V3-stage of WT (B104) and T2 *ZmWRKY82*-overexpressing maize strains were placed in propagation trays and watered until the soil moisture reached 80% of field capacity. Subsequently, the maize plants were subjected to 14 d of water withholding treatment until the soil moisture reached 35% of field capacity, and then subjected to 3 d re-watering treatment, maintaining soil moisture at 80% of field capacity. Soil moisture content was measured with a soil moisture meter.

For the ABA stress assay, maize seeds were germinated in an incubator at 28 °C for 2 d, and then treated with 10 μM ABA for 4 d. The root length was photographed using a Nikon D7000 camera (Nikon, Tokyo, Japan). All images were taken with a Nikon D7000 camera.

### 4.8. Detection of Physiological Indices

Following the method of Wu et al. (2025) [61], the SOD and POD activity and MDA content in the WT *Arabidopsis* and *ZmWRKY82*-overexpressing *Arabidopsis* were evaluated at 0 d and after 10 d of the water withholding treatment. Similarly, the activities of these enzymes were measured at 0 and after 7 d of water withholding in WT (B104) and *ZmWRKY82*-overexpressing maize lines. Fresh leaf samples (0.1 g) with uniform growth were homogenized in 1 mL extraction buffer to prepare the concentration of extract at 0.1 g/mL. The crude extracts were used to determine SOD and POD activity. Measurement of SOD activity was carried out by using the nitroblue tetrazolium (NBT) method [62]. Measurement of POD activity was carried out by employing the guaiacol assay [63]. Fresh leaf samples (0.1 g) with uniform growth were homogenized in 1 mL of 5% trichloroacetic acid (TCA) to obtain the concentration of extract at 0.1 g/mL for determining the MDA content. The MDA level was measured by reacting with thiobarbituric acid to form a colored complex [64].

### 4.9. Statistical Analysis

All experimental data were analyzed using one-way or two-way analysis of variance (ANOVA) in GraphPad Prism version 9.0. Three independent biological replicates were conducted for the experiment. A significance level of ** *p* < 0.01 was considered statistically highly significant.

## 5. Conclusions

In this study, we found that nuclear-localized *ZmWRKY82* has transcriptional activation potential and can specifically bind to the W-box sequence. *ZmWRKY82* can increase *Arabidopsis* and maize drought resilience by participating in the ROS signaling pathway and the ABA signaling pathway. These findings provide new insights into the function of *ZmWRKY82* in maize response to drought stress and provide an important theoretical foundation for cultivating drought-resistant varieties.

## Data Availability

All data generated or analyzed during this study are included in this published article and its Supplementary Materials files.

## Supplementary Materials

The following supporting information can be downloaded at https://www.mdpi.com/article/10.3390/plants14192943/s1, Figure S1: Sequence alignment and phylogenetic analysis of ZmWRKY82.; Figure S2: Detection of T3 generation transgenic *Arabidopsis thaliana* and T2 generation transgenic maize. Table S1: RT-qPCR Primers used in this study.

## Abbreviations

The following abbreviations are used in this manuscript:

TFs	Transcription factors
qRT-PCR	Quantitative real-time polymerase chain reaction
ABA	Abscisic acid
MeJA	Methyl jasmonate
SA	Salicylic acid
ETH	Ethephon
ROS	Reactive oxygen species
WT	Wild type
h	Hour
d	Day
V3	Third-leaf stage
CDS	Coding sequence
CLSM	Confocal laser scanning microscopy
SOD	Superoxide dismutase
POD	Peroxidase
MDA	Malondialdehyde
NBT	Nitroblue tetrazolium
TCA	Trichloroacetic acid
