# Supplementary material for: A WRKY Transcription Factor, ZmWRKY82, Conferred Enhanced Drought Stress Tolerance in Maize
Source: Plants (Basel). 2025 Sep 23;14(19):2943. doi: 10.3390/plants14192943 (PMC12526500; doi:10.3390/plants14192943)
Supplement: Supplementary file 1 [file plants-14-02943-s001.zip › Supplementary file Figures.pdf]

(a)

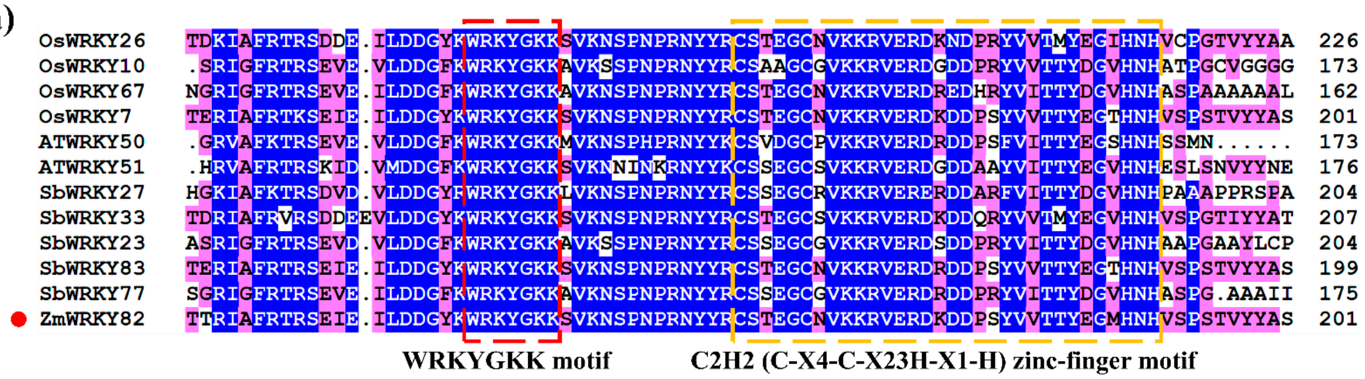

(b)

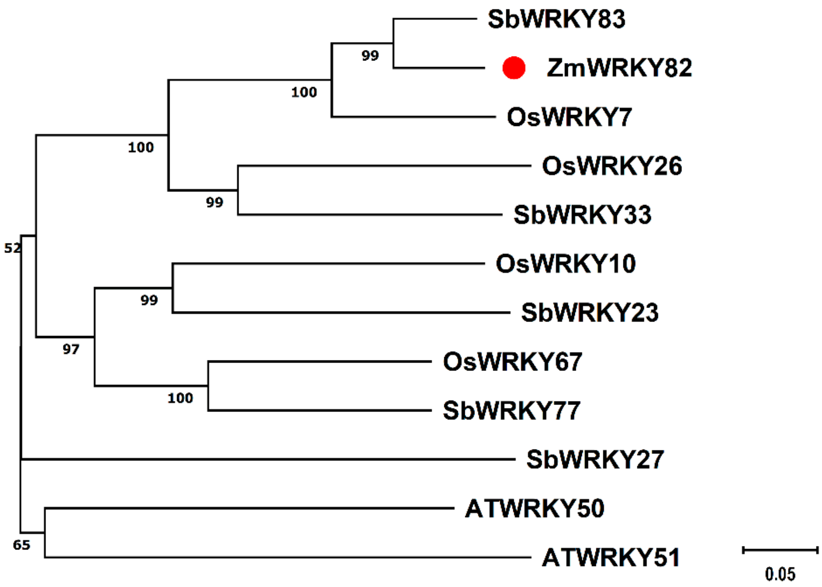

Figure S1. Sequence alignment and phylogenetic analysis of ZmWRKY82. (**a**) Alignment of amino acid sequences of ZmWRKY82. The conserved WRKY (WRKYGKK) motif and a (C-X_4-C-X_{23}-H-X_1-H) zinc-finger motif are indicated by boxes. (**b**) Phylogenetic analysis of ZmWRKY82 protein. The accession numbers were as follows: AT5WRKY50 (AT5G26170.1), ATWRKY51 (AT5G64810.1), OsWRKY7 (LOC_Os05g46020.1), OsWRKY10 (LOC_Os01g09100.1), OsWRKY26 (LOC_Os01g51690.1), OsWRKY67 (LOC_Os05g09020.1), SbWRKY23 (Sobic.003G037400.1), SbWRKY27 (Sobic.003G199400.1), SbWRKY33 (Sobic.003G276000.1), SbWRKY77 (Sobic.009G068900.1), SbWRKY83 (Sobic.009G212800.1), ZmWRKY82 (Zm00001d038843). The different background colors represent the similar degree of amino acid sequences. Blue: the similar degree of amino acid sequences is less than 100% and greater than or equal 75%. Pink: amino acid sequence similarity is less than 75% and greater than or equal to 33%. The red dot indicates the *ZmWRKY82* (Zm00001d040323).

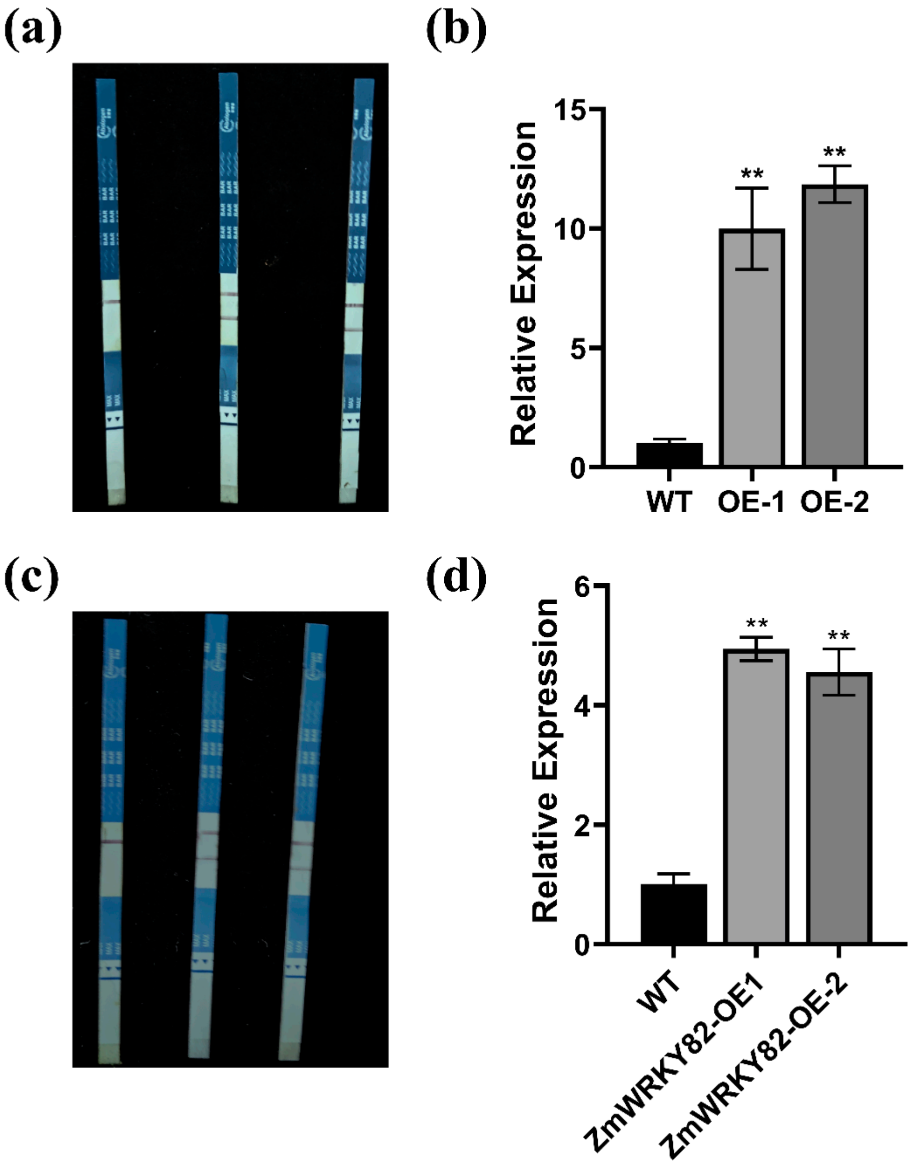

Fig. S2. Detection of T3 generation transgenic *Arabidopsis thaliana* and T2 generation transgenic maize. (**a**) Dipstick strips were used to detect transgenes of *Arabidopsis thaliana*. (**b**) Relative expression levels of ZmWRKY82 in the leaf of the transgenic lines OE1 and OE2. (**c**) Dipstick strips were used to detect transgenes of maize. (**d**) Relative expression levels of *ZmWRKY82* in the leaf of the transgenic lines ZmWRKY82-OE1 and ZmWRKY82-OE2.The analysis of significance compared with WT was performed using one-way ANOVA (** $p < 0.01$). Bars indicate the standard deviation of the mean. The experiment was performed using three biological replicates.
