# Supplementary figures and images for: A WRKY Transcription Factor, ZmWRKY82, Conferred Enhanced Drought Stress Tolerance in Maize
Source: Plants (Basel). 2025 Sep 23;14(19):2943. doi: 10.3390/plants14192943 (PMC12526500; doi:10.3390/plants14192943)

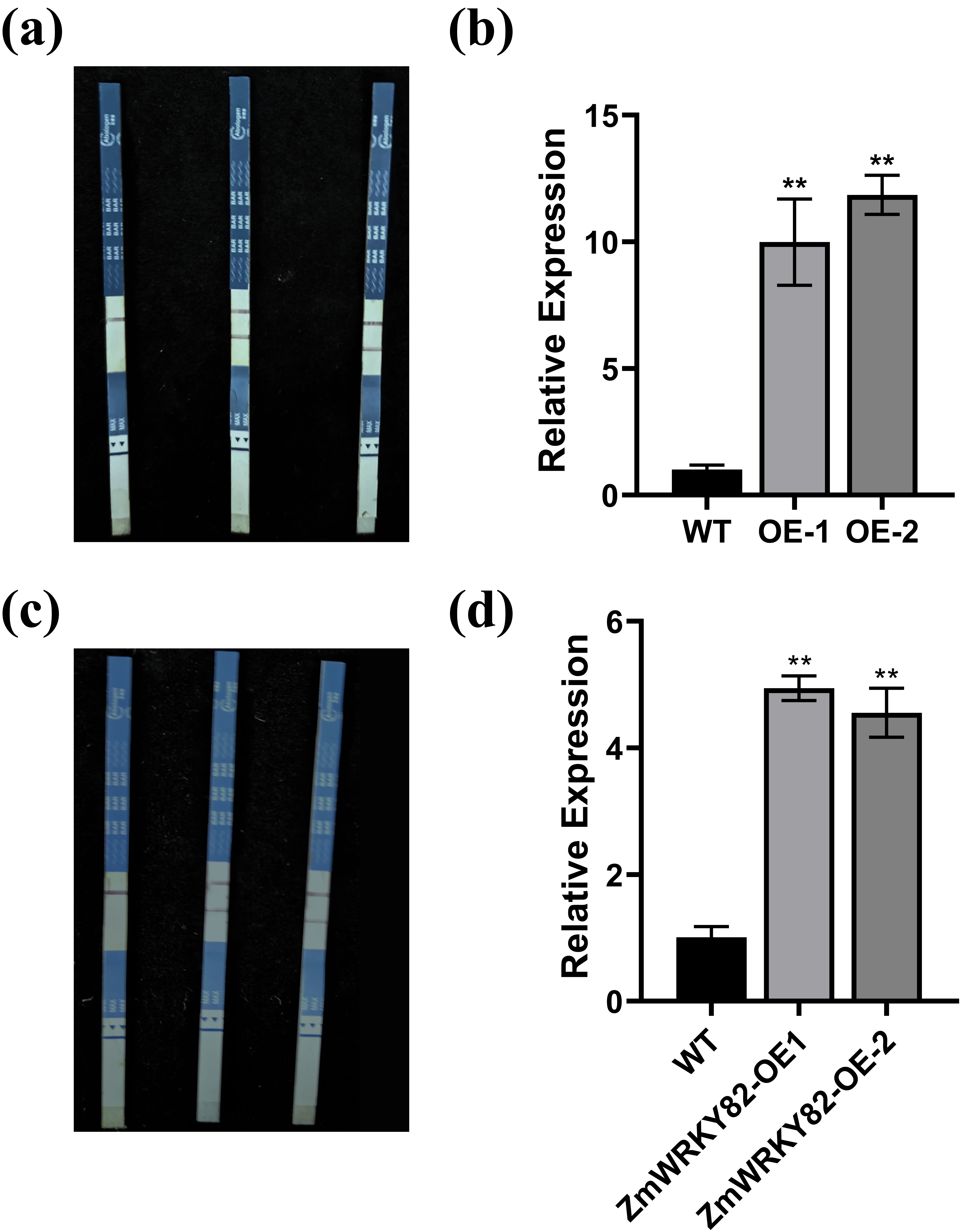

Supplement: Supplementary file 1 [file plants-14-02943-s001.zip › Figure S2.tif]
